# The Management of Plaque Psoriasis With Halobetasol and Tacrolimus Combination Therapy Versus Calcipotriol Monotherapy: A Case Report

**DOI:** 10.7759/cureus.52445

**Published:** 2024-01-17

**Authors:** Mohammed Z Sarwar, Nawaid H Khan, MIrza Masroor Ali Beg, Niloufer Mariam Javeed Ankolvi, Kudaibergen Osmonaliev

**Affiliations:** 1 Faculty of Medicine, Ala-Too International University, Bishkek, KGZ; 2 Microbiology, Ala-Too International University, Bishkek, KGZ; 3 Faculty of Medicine, Ala-Too Internatonal University, Bishkek, KGZ

**Keywords:** combination therapy, calcipotriol, plaque psoriasis, tacrolimus, halobetasol

## Abstract

Psoriasis is an inflammatory, immune-mediated, persistent, and multifactorial skin disease. Chronic plaque psoriasis is the most common clinical form of psoriasis. Pro-inflammatory cytokines play a primary role in the pathogenicity of this disease. Psoriasis is mainly diagnosed using clinical and dermoscopic examination of the cutaneous lesions, and skin biopsy is used in atypical cases. Psoriasis has no definitive cure. However, several topical agents are effective in managing mild and chronic cases. Combination therapy with these topical agents is more effective than with a single agent. We report a case of chronic plaque psoriasis in a 33-year-old man presenting with an itchy circumscribed, erythematous, scaly plaque, and a single cutaneous lesion covering >50% of both forearms and a few lesions on the back. The right forearm was treated with calcipotriol alone, whereas the left forearm was treated with a tacrolimus and halobetasol combination with emollients to be applied twice a day on both arms. We observed treatment responses for seven days with 24-hour intervals after each application. Combination therapy yielded a better response. In conclusion, topical treatment with a combination of halobetasol and tacrolimus is more effective compared to that with a single agent while being cost-effective and causing minimal adverse effects.

## Introduction

Psoriasis is an immune-mediated, inflammatory, chronic, recurrent dermatosis. Several environmental and genetic factors are associated with its pathogenicity. Psoriasis affects 2-3% of the general population globally [1]. Psoriasis has a multifactorial etiology. The most commonly observed clinical manifestation of psoriasis is chronic plaque psoriasis, which is typically localized to the extremities, trunk, scalp, and nails and occasionally affects the genitalia and the anal region [2]. Previous studies have reported elevated levels of proinflammatory cytokines such as TNF-alpha, IL-6, IL-17, and others both in localized lesion regions and blood in patients with psoriasis. Both local and systemic inflammation play a crucial role in the pathophysiology of psoriasis [3]. While no standard diagnostic test exists, diagnosis is usually made by clinical observation of the cutaneous lesions, and a skin biopsy is performed in cases with an atypical manifestation. Although a definitive cure for psoriasis is lacking, various treatment modalities are used to manage chronic psoriasis, such as topical agents, phototherapy, systemic therapy, and biological agents. Calcipotriol is a vitamin D3 analogue and an antiproliferative agent that reduces the abnormal proliferation of keratinocytes associated with psoriasis and induces normal cell differentiation that normalizes epidermal growth. Halobetasol is a corticosteroid with anti-inflammatory properties [1,2]. Tacrolimus (macrolide) is another effective treatment option. It is extracted from the bacterium *Streptomyces tsukubaensis *and acts by inhibiting calcineurin, which suppresses both CD4 and CD8 T-cell activation [4].

Previous studies have shown the combination of tacrolimus, calcipotriol, and halobetasol to be more effective in managing mild to chronic plaque psoriasis compared to monotherapy with any of these agents, attributing the superior efficacy of the combination therapy to the likely synergistic immunological and fibrinogenic effects [5, 6].

Herein we report an observational case study of plaque psoriasis manifesting lesions on bilateral forearms. The patient received calcipotriol monotherapy on one arm and a combination of tacrolimus and halobetasol on the other, and the response to both treatments was observed for one week.

## Case presentation

A 33-year-old man with a five-year history of psoriasis presented with an itchy, circumscribed, erythematous, scaly plaque covering more than 50% of the forearm on both forearms, along with a few lesions on the back. The patient reported lesion flare-ups in stressful conditions and had no family history of psoriasis. Dermoscopic examination showed red dots with regular distribution and white scales.

At the time of presentation, the patient was not under any topical or systemic treatment but was regularly using emollients. Initially, for two years, the patient was on methotrexate (MTX) (first year, first three months MTX 7.5 mg weekly with a tapering dose (5 mg weekly for 15 days, 2.5 mg for 15 days) and then in the second year again the same MTX regimen was followed and stopped). The patient had not been on MTX for three years and had not used any specific medicine except emollients. The patient had a history of using methotrexate orally for two years, with 90% resolution of the lesions and a Psoriasis Area and Severity Index score of 2.

**Figure 1:**
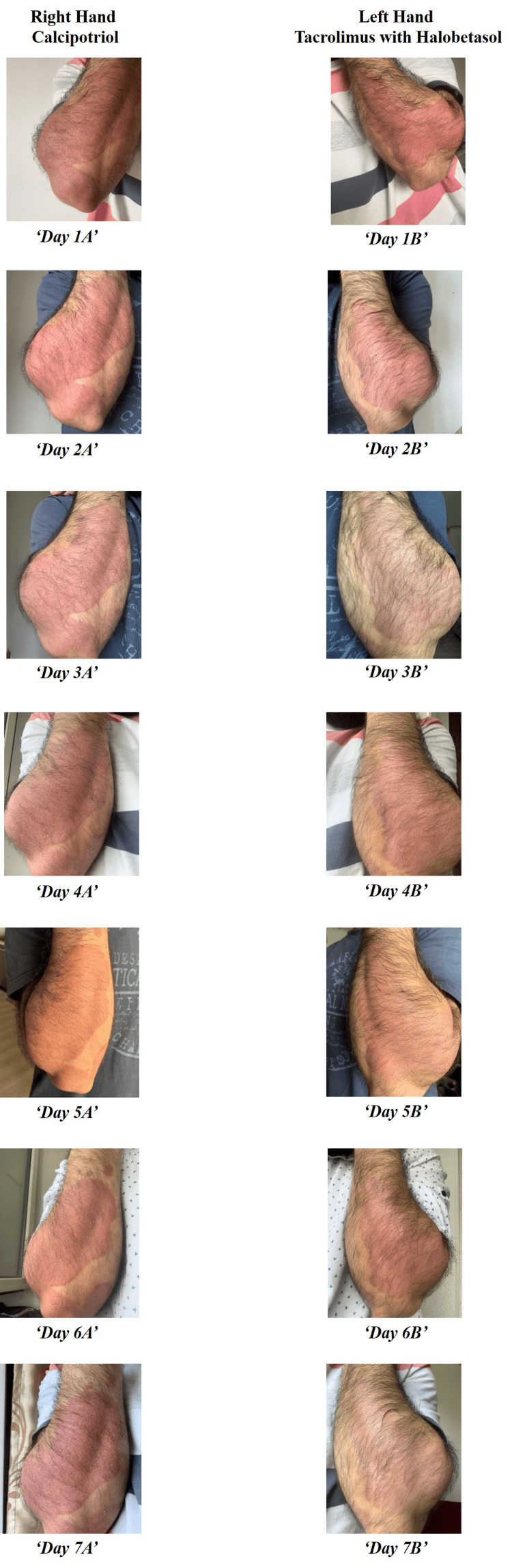
Depiction of treatment response for one week with different therapies daily: (a) right-hand treatment with calcipotriol; (b) left-hand treatment with halobetasol and tacrolimus together.

For the most recent episode of relapse, we prescribed topical calcipotriol (0.005%) monotherapy for the right forearm lesion and a combination of tacrolimus (0.03%) and halobetasol (0.05%) for the left forearm lesion, with emollients to be applied twice a day bilaterally. We then observed responses to both treatments for seven days, with 24-hour intervals between each observation (Figure 1a, 1b). On the first day of observation after treatment, we observed a minor resolution of the lesion on the left forearm that received the combination therapy. However, the right forearm lesion receiving monotherapy did not show noticeable resolution. Furthermore, on the third day of observation, the treatment response was significantly better on the left forearm lesion (combination therapy) than on the right forearm lesion. However, we did not measure the lesion size before and after treatment.

On the seventh day after treatment initiation, a significant resolution was observed on the left forearm lesion that received the combination therapy. We then administered the combination therapy on bilateral forearm lesions for one more week until further follow-up. The observation and details of the treatment response are shown in Figure 1a,b.

## Discussion

Psoriasis is an immune-mediated, inflammatory disorder resulting from the dysregulation of both innate and adaptive immune responses and altered keratinocyte proliferation and function. Plaque psoriasis is a chronic clinical manifestation of psoriasis associated with recurrent relapses. A prolonged increase in proinflammatory cytokine levels plays a critical role in effecting chronic inflammation and subsequent comorbidity [3,4,7]. Non-treatment may result in complications such as sepsis, high-output cardiac failure, anemia, and malabsorption [8]. Several treatment options for the management of psoriasis are currently available, such as biological therapy, psoralen plus ultraviolet-A radiation therapy, and systemic therapy with immunosuppressive agents [8, 9]. However, topical management remains the most cost-effective treatment option with no or minimal adverse effects, such as local irritation at the application site. However, prolonged use is not recommended due to the risk of skin atrophy, particularly with potent corticosteroids like halobetasol [5,6,10]. Although treatment with vitamin D3 analogues has been found to be most effective, the treatment response is not satisfactory in the case of monotherapy. However, combination therapy with vitamin D3 analogs and clobetasol/halobetasol has yielded satisfactory results [11]. Our case showed that combination therapy with halobetasol and tacrolimus also yields satisfactory results. As tacrolimus is a calcineurin inhibitor that modulates the immune response, and halobetasol, which is a potent topical corticosteroid with anti-inflammatory effects, the combination of these two agents may have a synergistic effect, providing a more comprehensive suppression of inflammation and immune responses.

## Conclusions

Our case demonstrates that the combination of halobetasol and tacrolimus yields better results than monotherapy with calcipotriol, offering low cost and minimal side effects in the treatment of plaque psoriasis. While the use of potent steroids provides quicker resolution, it is associated with a high atrophy rate. Halobetasol is a potent corticosteroid, and hence its prolonged use can cause adverse effects. However, our case indicates that prolonged use of topical tacrolimus and calcipotriol can be administered with mild to no adverse effects.
